# CEACAM1 in Liver Injury, Metabolic and Immune Regulation

**DOI:** 10.3390/ijms19103110

**Published:** 2018-10-11

**Authors:** Andrea Kristina Horst, Sonia M. Najjar, Christoph Wagener, Gisa Tiegs

**Affiliations:** 1 Institute of Experimental Immunology and Hepatology, Center for Experimental Medicine, University Medical Center Hamburg-Eppendorf, Martinistraße 52, 20251 Hamburg, Germany; g.tiegs@uke.de; 2 Department of Biomedical Sciences, Heritage College of Osteopathic Medicine, Irvine Hall, 1 Ohio University, Athens, OH 45701-2979, USA; najjar@ohio.edu; 3 The Diabetes Institute, Heritage College of Osteopathic Medicine, Irvine Hall, 1 Ohio University, Athens, OH 45701-2979, USA; 4 University Medical Center Hamburg-Eppendorf, Martinistraße 52, 20251 Hamburg, Germany; wagener@uke.de

**Keywords:** CEACAM1, immune checkpoint receptor, liver disease, insulin clearance

## Abstract

Carcinoembryonic antigen-related cell adhesion molecule 1 (CEACAM1) is a transmembrane glycoprotein that is expressed on epithelial, endothelial and immune cells. CEACAM1 is a differentiation antigen involved in the maintenance of epithelial polarity that is induced during hepatocyte differentiation and liver regeneration. CEACAM1 regulates insulin sensitivity by promoting hepatic insulin clearance, and controls liver tolerance and mucosal immunity. Obese insulin-resistant humans with non-alcoholic fatty liver disease manifest loss of hepatic CEACAM1. In mice, deletion or functional inactivation of CEACAM1 impairs insulin clearance and compromises metabolic homeostasis which initiates the development of obesity and hepatic steatosis and fibrosis with other features of non-alcoholic steatohepatitis, and adipogenesis in white adipose depot. This is followed by inflammation and endothelial and cardiovascular dysfunctions. In obstructive and inflammatory liver diseases, soluble CEACAM1 is shed into human bile where it can serve as an indicator of liver disease. On immune cells, CEACAM1 acts as an immune checkpoint regulator, and deletion of *Ceacam1* gene in mice causes exacerbation of inflammation and hyperactivation of myeloid cells and lymphocytes. Hence, hepatic CEACAM1 resides at the central hub of immune and metabolic homeostasis in both humans and mice. This review focuses on the regulatory role of CEACAM1 in liver and biliary tract architecture in health and disease, and on its metabolic role and function as an immune checkpoint regulator of hepatic inflammation.

## 1. Introduction

Carcinoembryonic antigen-related cell adhesion molecule 1 (CEACAM1) is a member of the CEA family of highly glycosylated cellular adhesion molecules, which belongs to the immunoglobulin superfamily [1,2,3]. CEA proteins are built from a highly conserved modular set of N-terminal variable-like and constant-like immunoglobulin domains that mediate *cis* or *trans* homophilic or heterophilic adhesion [4,5]. Amongst other members of the CEA family, human *CEACAM1* gene undergoes the most extensive alternative splicing, giving rise to 12 splice variants [6,7]. The two major isoforms of CEACAM1 in humans and mice contain four extracellular domains and either a long or a short cytoplasmic tail (CEACAM1-4L or -4S, respectively) that can differentially control cellular activation, differentiation, migration/invasion, and proliferation. The other commonly expressed pair of splice variants of the gene contains two extracellular domains with either a long or short cytoplasmic tail (CEACAM1-2L or -2S, respectively). In the CEACAM family, CEACAM1-L is unique in that its cytoplasmic tail contains two immunoreceptor tyrosine receptor-based inhibition motifs (ITIMs; consensus sequence: S/I/V/LxYxxI/V/L) in human CEACAM1-L, and two immunoreceptor tyrosine-based switch motifs [ITSM; consensus sequence: TxYxx(V/I)] in rodent CEACAM1-L. These structural features indicate that CEACAM1-L transmits inhibitory signals upon ligand or co-receptor engagement [7]. CEACAM1-L contains two tyrosine residues that are substrates for Src-kinase and can be dephosphorylated by the inhibitory SH2-containing tyrosine phosphatases 1 and 2 (SHP-1, SHP-2 [8]). The short isoform of CEACAM1 (CEACAM1-S) can bind to calmodulin, tropomyosin, globular actin, annexin II, and polymerase delta interacting protein p38 (PDIP38), and is phosphorylated by protein kinase to regulate cytoskeletal dynamics [9,10,11,12,13]. The different CEACAM1-isoforms are most frequently co-expressed by the same cell and their relative expression levels determine the outcome of cellular signaling [5,14,15].

**Figure 1:**
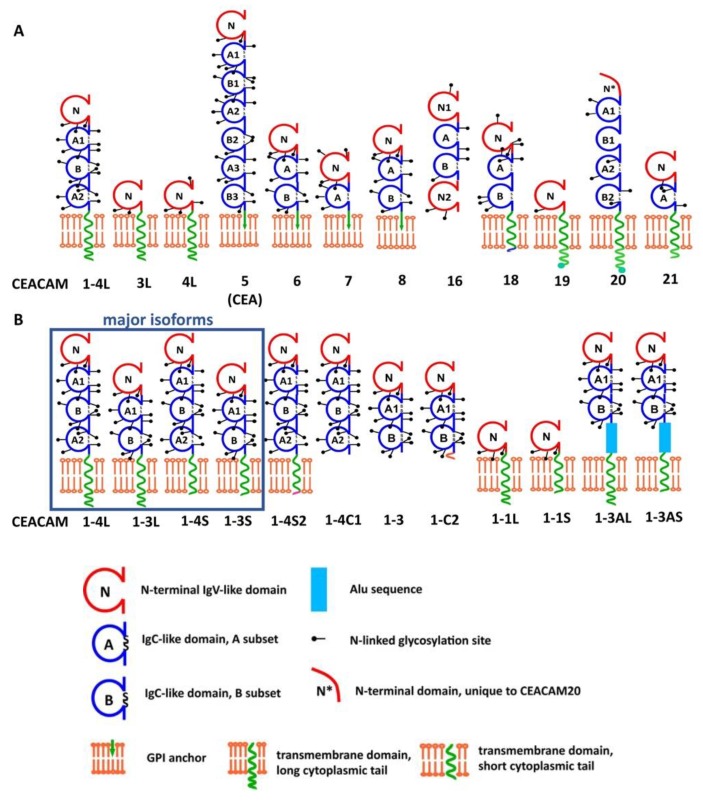
Structural representation of human CEACAM proteins and CEACAM1 splice variants. (**A**) In humans, there are 12 different CEACAM genes that encode functional proteins: CEACAM1, CEACAM3, CEACAM4, CEACAM5 (CEA), CEACAM6, CEACAM7, CEACAM8, CEACAM16, and CEACAM18-CEACAM21. Amongst these, CEACAM16 is expressed as a soluble protein. CEACAM1L, 3L and 4L as well as CEACAM18-21 possess a transmembrane anchor with a cytoplasmic tail (represented by L), whereas CEACAM5-8 are GPI-linked. (**B**) Schematic representation of the 12 CEACAM1 protein isoforms as products of alternative splicing of the human *CEACAM1* gene: The most prominent and best-studied CEACAM1 isoforms are highlighted in a blue box. They either comprise a long (L) or a short cytoplasmic tail (S) and four or three extracellular immunoglobulin-like domains (1-4 or 1-3, respectively). Amongst the 12 isoforms, further 4-domain variants are found with a modified short cytoplasmic tail (CEACAM1-4S2) or a soluble isoform (CEACAM1-4C1). Additional soluble isoforms include CEACAM1-3 and CEACAM1-C2. The membrane-bound CEACAM1-1L and CEACAM1-1S as well as CEACAM1-AL and CEACAM1-AS only comprise one extracellular domain. Their functions remain elusive. Further details and hyperlinks to protein databases are found on www.carcinoembryonic-antigen.de; a full list of genes encoding CEACAM proteins in humans and rodents can be found in [29]. Adapted in modified form from www.carcinoembryonic-antigen.de, with permission.

Discovery and characterization of what is now called CEACAM1 is best summarized as a convergence of paralleling evolutionary threads that have described CEACAM1 in different biological contexts as non-specific cross-reacting antigen with antibodies directed against CEA, biliary glycoprotein-I (BGP-I), C-CAM1 (a calcium-dependent cell-cell adhesion molecule from rat hepatocellular membranes), ecto-ATPase from hepatocellular membranes [3,16], gp110 (a “transformation-sensitive glycoprotein” [17]), pp120/ecto-ATPase/HA4 [a substrate of the insulin receptor [18,19], MHVR1 (a murine hepatitis virus receptor [20,21]), CGM1 (the CEA gene family member 1 in the mouse [22,23,24]), and CD66a (protein of the cluster of differentiation (CD) antigen on human neutrophils [25,26,27,28]). This heterogenous nomenclature was revised to define individual CEACAMs unanimously as members of the CEA family of cell adhesion molecules with the assignment of individual CEACAM numbers [29] (Figure 1).

CEACAM1 is expressed on epithelia, endothelia and leukocytes, but is absent from skeletal muscle myocytes and cartilage. The protein is both structurally and functionally conserved in humans and rodents and shares the same tissue expression pattern [30,31,32,33,34]. In mice, four major CEACAM1 isoforms exist that comprise the CEACAM1-4L and CEACAM1-4S isoforms, as well as two isoforms with two extracellular domains and either a long or a short cytoplasmic tail (CEACAM1-2L and CEACAM1-2S; www.cea-homepage.de). Additionally, a strain-specific allelic variant (*Ceacam1b*; SJL/J mice) has been discovered, whereas C57BL/6J, Sv129Sv/J, and Balb/C mice express the common *Ceacam1a* allele [21,35]. 

## 2. Discovery of CEACAM1 in Patients with Liver Disease

CEACAM5, the carcinoembryonic antigen, was originally detected in the search for tumor-specific antigens in the colon, and later as a normal tissue antigen in the fetal and adult digestive tract [36,37,38,39]. CEACAM1 was first described as a biliary glycoprotein (BGP) and a CEA-like substance in human hepatic bile. Svenberg et al. [40] discovered that components of human bile from patients with biliary obstruction exhibited cross-reactivity with anti-CEA (CEACAM5) antisera in the absence of gastrointestinal malignancies. Purification and partial characterization of this *biliary glycoprotein 1* (BGP-I) from hepatic bile enabled the first partial characterization of what was later re-named CEACAM1 [29,40]. Specific association of BGP-I/soluble CEACAM1 expression in the serum of patients with obstructive liver disease was found. Indeed, BGP-I serum level was considered a surrogate marker in various liver diseases (chronic hepatitis, alcoholic cirrhosis, primary biliary cirrhosis) with comparable validity to established markers such as gamma glutaryl transferase (GGT) and alkaline phosphatase (ALP). For instance, serum BGP-I is five- to tenfold higher in patients with cholestasis by comparison to 0.2–0.9 mg/L in healthy subjects and correlates significantly (*p* < 0.0005) with the elevation of GGT levels. In primary biliary cirrhosis, BGP-I serum levels correlate significantly with ALP (*p* < 0.005) [41]. In addition, soluble CEACAM1 is strongly increased in the serum and urine of rats with liver disease [42]. Interestingly, CEA is predominantly present in the serum of patients with cholangioma and cholangiocarcinomas compared to patients with cholelithiasis, and in patients who have undergone non-curative surgery, whereas CEACAM1 is detectable in all biliary specimen without discrimination between the presence or absence of malignant disease [43]. 

However, these putatively differential indications for diagnosis and prediction of CEA and CEACAM1 expression in the serum of patients with a spectrum of hepatobiliary diseases have not been pursued further, owing largely to the lack of specific monoclonal antibodies that could reliably distinguish between CEA, NCAs and the biliary glycoprotein I (BGP-I). In fact, the first monoclonal antibodies that could exclusively detect CEACAM1 were developed by Wagener’s group against BGP-I in human bile [44,45]. Cloning of CEACAM1 cDNA revealed more than 80% homology at the nucleotide level with corresponding domains in CEA and the former NCAs. Only the CEACAM1-specific A2-domain exhibits approximately 56% homology with other CEA family member domains [2]. In order to identify the source of cells producing BGP-I in human livers, immunofluorescence studies were undertaken that localized BGP-I to bile canaliculi, the lumen of larger bile ducts, and the surface of the gall bladder mucosa [46]. This expression pattern of human CEACAM1 has also been confirmed in rodents [31,47,48,49,50]. 

### 2.1. CEACAM1 in Hepatocyte Adhesion, Differentiation, and in Liver Regeneration 

**Figure 2:**
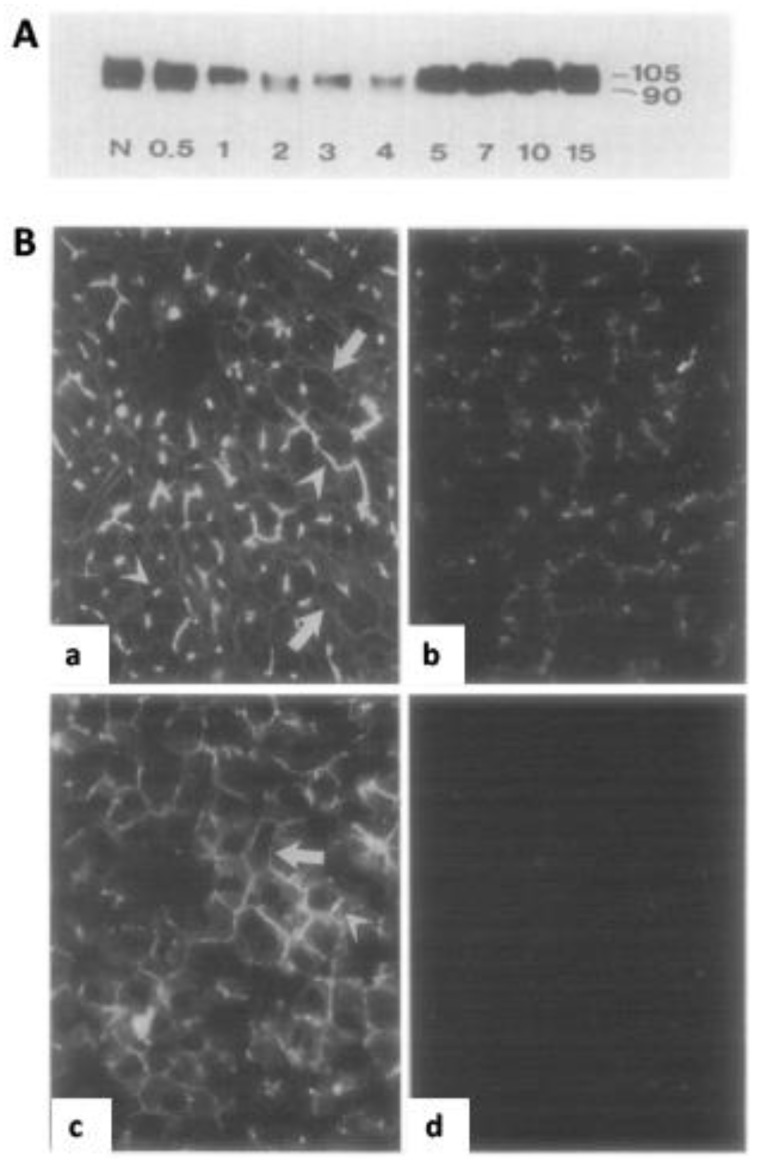
Dynamic expression of CEACAM1 in regenerating rat livers after partial hepatectomy. (**A**) A Western Blot showing CEACAM1 expression before (N) and after partial hepatectomy at the indicated timepoints (days) in rats. The decline in CEACAM1 expression starts 1 day after the procedure and returns back to near normal levels approximately after 7–10 days. (**B**) Analysis of CEACAM1-expression by indirect fluorescence (white signals) in a healthy rat liver (**a**), 48 h (**b**), and 15 days (**c**) after partial hepatectomy. CEACAM1 is expressed on all membranes of the hepatocytes with especially strong staining present at the apical surface of the hepatocytes. Weaker staining is present on the hepatocellular surface facing the sinusoidal space (a,c: white arrows). (**d**) negative control. (**A**,**B**) reprinted with modifications from [53] with permission from Elsevier.

The prominent expression of CEACAM1 on rat hepatocellular membranes and its role in promoting cellular aggregation of hepatocytes, prompted the initial demonstration that it functions as an intercellular adhesion molecule [51,52]. Hence, this candidate cell adhesion molecule was named cell-CAM105 based on its apparent molecular mass and reflecting its calcium-dependent homophilic hepatocellular and liposome aggregation formation [47,51,52]. Studies in the fetal and regenerating rat liver revealed that CEACAM1 is first expressed in fetal liver on day 16 after gestation in the rat [53]. In mice, CEACAM1 is detected as early as day 10.5 after gestation in the developing gut [30], and continuously increases until 3 weeks after birth at which point adult levels of hepatic CEACAM1 expression are reached [53]. Of note, the expression of both major CEACAM1 isoforms (CEACAM1-S, CEACAM1-L) increases proportionally after birth, indicating that CEACAM1 is involved in hepatocyte differentiation and is a hallmark molecule inducing epithelial differentiation. This hypothesis was corroborated by the description of the dynamics of CEACAM1 expression after partial hepatectomy in rats (Figure 2): CEACAM1 is initially down-regulated on the surface of hepatocytes, when cell proliferation peaks, but it is then restored back to steady-state levels 15 days post-partial hepatectomy, at which point the liver is reconstituted ad integrum in healthy individuals [53,54,55]. Notably, after partial hepatectomy in healthy individuals, the liver regains its original mass within 7–10 days after this procedure [54,55]. Hence, the partial hepatectomy model is frequently used for the analysis of well-coordinated cellular proliferation and differentiation in tissue regeneration, which is naturally self-limiting.

CEACAM1 expression is regulated by the hepatocyte nuclear factor 4α (HNF4α), and liver-specific transcription factor in humans and rodents, amongst others [56,57,58,59]. HNF4α coordinates the expression of cellular adhesion proteins in the developing mouse liver, as demonstrated by junction dysformation in mice with genetic deletion of HNF4α [60]. Loss of HNF4α causes about three to fourfold downregulation of *Ceacam1* transcript expression and totally abolishes the expression of E-cadherin and claudin-1 in embryos [60]. In adult mice, severe deficits in liver homeostasis occur upon loss of HNF4α expression, such as hyperproliferation of hepatocytes and disturbed lipid homeostasis, consistent with the role of HNF4α in regulating the expression of several genes involved in lipid and glucose metabolism [56,61,62]. In addition, characterization of the rat *Ceacam1* promoter revealed binding sites for other liver-specific factors, such as hepatonuclear factor 5 (HNF5), CCAAT/enhancer binding protein (C/EBP), and hepatonuclear factor 1 (HNF1), as well as for glucocorticoids, cAMP-response element binding protein (CREB) and activator protein 1 and 2 (AP-1, AP-2, [58]). In hepatocytes, CEACAM1 is expressed as two alternative spliced isoforms that differ in their adhesive properties, with the long isoform (CEACAM1-L) mediating direct homophilic cell-cell adhesion, and the short isoform (CEACAM1-S) being responsible for fine-tuning of the adhesive properties of the L-form [32]. This points to a function for CEACAM1 in tissue architecture organization. Consistently, CEACAM1’s expression is absent in transplantable hepatocellular carcinomas of the rat [17]. This prompted the hypothesis that coordination of cell growth and maintenance of epithelial polarity and differentiation depends on CEACAM1 expression. In addition, localization of CEACAM1 to cell-cell contacts in epithelial cells requires its long cytoplasmic tail, whereas its targeting to the apical surface depends on the short cytoplasmic tail [63,64]. Therefore, CEACAM1 has been considered a tumor suppressor and a regulator of epithelial cell polarity, and that significant alterations of its expression are features of malignancy [65]. Furthermore, CEACAM1 has been described as a “transformation-sensitive protein”, the loss of which is indicative of cellular transformation or de-differentiation of hepatocytes [17].

### 2.2. CEACAM1 in Hepatocellular Carcinomas

In general, dysregulation of CEACAM1 expression is frequently observed during malignant progression and invasion of various carcinomas [66,67]. However, although tumor-suppression has been ascribed to CEACAM1 in a variety of tumors such as mammary, prostate and colonic adenocarcinomas, increased invasion has also been assigned to elevated CEACAM1 expression in some others, including pancreatic, lung (NSCLC) and thyroid adenocarcinomas and melanomas. In these tumors, CEACAM1 expression is associated with poor prognosis and invasion. In the colon, focal loss of CEACAM1 is found in early tumorigenesis, and it appears to be upregulated during invasion and metastasis of advanced disease ([15,66,67] and references therein). In general, it is proposed that inhibition of malignant growth depends on the long cytoplasmic domain of CEACAM1 and that growth control requires phosphorylation of the tyrosine residues within its ITIM motifs. These tyrosine residues and their interaction with the inhibitory phosphatase SHP-1 blunt growth-promoting signals instructed by growth factor receptor tyrosine kinase signaling and downstream Src-kinase activity [8,68,69]. In human mammary gland formation, both the short and long isoforms of CEACAM1 are associated with lumen formation, and the expression of CEACAM1-4S in human mammary carcinoma cells re-introduces normal mammary gland differentiation and abolishes the malignant phenotype [70,71]. Hence, CEACAM1 isoforms can exert divergent roles in tumorigenesis and malignancy.

With regards to human hepatocellular carcinomas (HCC), CEACAM1 expression is predominantly maintained in the carcinomas of the well-differentiated type, with the bile canaliculi and the apical domains of pseudoglands expressing CEACAM1. In contrast, in poorer differentiated tumor areas, the expression of membranous CEACAM1 is weaker [72]. This was subsequently confirmed in later studies that showed a downregulation of membranous CEACAM1 expression and/or a predominant intracytoplasmic localization of CEACAM1 in HCC in correlation with higher histological grade. Consistently, loss of CEACAM1 expression is more frequently observed in tumor HCC specimens with Edmondson-Steiner-Grades III or IV, compared to Edmondson-Steiner Grades I and II. Congruently, loss of CEACAM1 expression is significantly associated with tumor size, multiplicity of tumor nodules, formation of satellite nodules, and capsular and vascular invasion (portal vein), all of which are adverse to patient survival [73,74]. 

However, few studies have investigated the isoform-specific impact of CEACAM1 expression or its subcellular localization on malignancy and prognosis in HCC. In recent years, it has become evident that loss of apical membranous CEACAM1 expression with a concomitant shift to intra-cytoplasmic localization corresponds to decreased relapse-free survival of HCC after liver transplantation [74]. These specimens with loss of membranous versus prominent bile canalicular staining of CEACAM1, exhibited an inverse correlation of CEACAM1 expression with increased microvascular density, which was correlated with an unfavorable high histological grade [75]. With regard to whether CEACAM1 isoforms impact differentially the prognosis of HCC, we have found that a predominant expression of CEACAM1-L constitutes a risk factor for HCC recurrence, and that predominant CEACAM1-S expression inhibits recurrence of HCC [76]. Thus, CEACAM1 is an independent prognostic factor for disease recurrence which is qualitatively comparable to prognosis estimation derived from assessment of tumor size ≥5 cm, or the presence of multinodular HCCs. In vitro, it was shown that Smad (small mothers against decapentaplegic) adaptor protein β2SP binds to the long cytoplasmic domain of CEACAM1-L and promotes nuclear translocation of Smad3 to activate transforming growth factor β (TGFβ)-mediated signaling [76]. This may indicate that CEACAM1-L supports the epithelial mesenchymal transition. Similar to HCC, expression of CEACAM1 was found at the invasive front of melanomas and showed significant prediction of metastatic disease [77,78,79]. The association of CEACAM1-L with αvβ3 integrin promotes cellular invasion [77]. These invasion-associated functions of CEACAM1 depend on the presence of an intact Tyr488 in its long cytoplasmic domain [77]. 

### 2.3. CEACAM1 in Metabolism: A Role in Promoting Insulin Clearance 

CEACAM1, which had been known as pp120/HA4/ecto-ATPase, was identified as a substrate of the insulin receptor (IR) tyrosine kinase in liver, but not in other insulin-sensitive tissues like skeletal muscle and adipose tissue [18,19,80,81,82]. Recombinant CEACAM1 was found to be phosphorylated by the IR tyrosine kinase in stably transfected cells and in a cell-free system with hepatocellular membrane fractions incubated with solubilized insulin receptors, as well as in hepatoma cells [81]. Contrary to other insulin receptor substrates, CEACAM1 is not phosphorylated by the insulin-like growth factor receptor (IGF-IR) upon insulin-like growth factor 1 (IGF-1) binding, consistent with the fact that CEACAM1 downregulates the mitogenic activity of insulin but not IGF-1 [83]. This downregulatory function of phosphorylated CEACAM1 on cell proliferation in response to insulin is mediated by its binding to and sequestering the SH2-containing adapter protein (Shc), an event that reduces coupling of the growth factor receptor-bound protein 2 (Grb2)/rat sarcoma (Ras) GTPases/MAPK pathways to the receptor [84,85,86].

**Figure 3:**
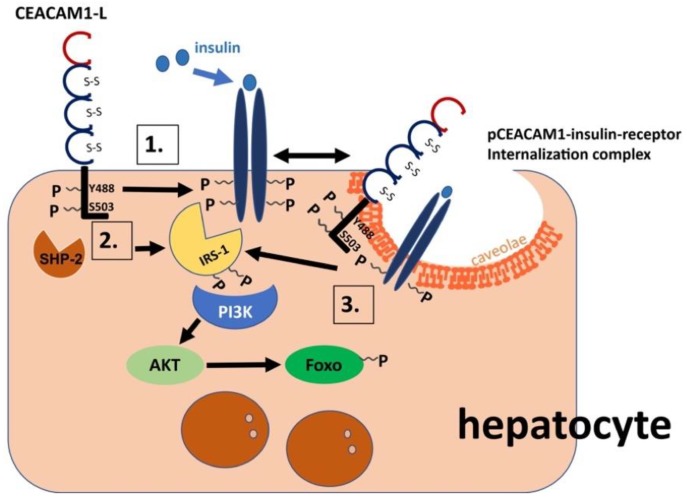
Regulation of insulin clearance by CEACAM1-L. (1.) Insulin binding to the insulin receptor (IR) induces its autophosphorylation and the phosphorylation of downstream substrates such as the insulin receptor substrate-1 (IRS-1) and CEACAM1-L. Phosphorylation of CEACAM1-L on Tyr488, which requires an intact Ser503 residue, mediates its indirect association with IR to increase the rate of endocytosis of the insulin-insulin receptor complex into clathrin pits/vesicles of the hepatocytes, facilitating subsequent insulin degradation and clearance. (2.) Phosphorylated CEACAM1 can bind to SHP-2 to sequester the phosphatase and subsequently, mediate more sustained phosphorylation of the IRS-1/PI3K/AKT pathway that transduces the metabolic effects of insulin, such as inhibiting gluconeogenesis (via phosphorylating and nuclear exclusion of forkhead box protein O1 (Foxo1), among others [86,93,94]). The physiologic correlation of this CEACAM1 expression is underlined by the negative effect of Foxo1 activation on *Ceacam1* expression [95]. Furthermore, CEACAM1-L–mediated IR internalization via clathrin-coated pits (3.) enhances the exposure of the insulin receptor tyrosine kinase to its endogenous substrates (IRS-1), supporting sustained IRS-1/PI3K/AKT signaling until insulin is degraded and removed. Single-headed black arrows indicate a chain of phosphorylation/dephosphorylation events by the indicated insulin receptor tyrosine kinase (IR), Ser/Thr kinases (PI3K/AKT), and SHP-2 phosphatase. Double-headed black arrows denote the physical (indirect) interaction between phosphorylated-CEACAM1-L (pCEACAM1) and the IR to stabilize the insulin-receptor endocytosis complex and increase its uptake into the endocytotic vesicles.

Site-directed mutagenesis indicates that CEACAM1 is phosphorylated on the conserved Tyr 488 (in the rat sequence) and that this requires an intact Serine 503 residue in the cytosolic tail [19]. Moreover, this phosphorylation is regulated by Tyr1316 in the C-terminus domain of the insulin receptor β subunit (IRβ) that is not conserved in IGF-1R [83,87]. Phosphorylation of CEACAM1 is also regulated by SHP-1 and SHP-2 phosphatases that bind to phosphorylated Tyr488 [88,89,90,91]. CEACAM1 binding to SHP-2 sequesters it to reduce its binding to insulin receptor substrate-1 (IRS-1), leading to sustained phosphorylation and prolonging insulin signaling along the IRS-1/Akt pathway [92]. The interaction between CEACAM1-L and the insulin receptor is summarized in Figure 3.

The kinase activity of IR is mandatory for receptor-mediated insulin endocytosis via clathrin coated pits/vesicles, which when followed by degradation, constitute the molecular basis of insulin clearance [96] that occurs primarily in the liver and to a lower extent in the kidney [97]. Thus, the tyrosine phosphorylated downstream targets of insulin constitute a critical component of the insulin signaling pathway that regulates insulin clearance. Hepatic insulin removal clears up to 80% of insulin released from pancreatic β cells into the portal circulation [97] to regulate the overall blood level of insulin and subsequently, its action. That insulin signaling in the liver is required to mediate systemic insulin sensitivity has been demonstrated by mice with conditional null mutation of the IR in the liver (LIRKO) [98]. These mice showed that loss of insulin signaling in hepatocytes leads to severe primary hepatic insulin resistance, glucose intolerance and chronic hyperinsulinemia, in part caused by impaired insulin clearance [99].

Interestingly, CEACAM1-L shares a homologous amino acid sequence with adaptins that anchor proteins in clathrin-coated pits [87]. Consistently, CEACAM1-L was found to associate with the low dominant high-affinity isoform A of the insulin receptor that promotes receptor-mediated insulin endocytosis and degradation in hepatocytes [19,84,87]. Studies on the mechanism of CEACAM1-mediated insulin action are amongst the first reports describing isoform-specific functions for alternative splice variants of the CEACAM1 protein [18,32]. As stated above, Ser503 is critical for basal phosphorylation by serine/threonine kinases and its substitution for Alanine (Ser503Ala) is sufficient to compromise insulin-stimulated Tyr488 phosphorylation in response to insulin in a dominant-negative manner [19,100]. L-SACC1 mice with transgenic liver-specific overexpression of the phosphorylation-deficient dominant-negative Ser503Ala mutant under the control of apolipoprotein A1 promoter [101] develop impaired insulin clearance, which causes chronic hyperinsulinemia and secondary systemic insulin resistance, glucose intolerance and hepatic steatosis [101]. In contrast, mice with global deletion of SHP-1 exhibit enhanced insulin clearance that results from CEACAM1 hyperphosphorylation and its constitutive activation in terms of promoting insulin internalization [102]. Collectively, these studies highlight the key role of CEACAM1 in regulating insulin sensitivity by promoting insulin clearance. 

### 2.4. CEACAM1: Implication in Non-Alcoholic Fatty Liver Disease 

Non-alcoholic fatty liver disease (NAFLD) and its progressive form, non-alcoholic steatohepatitis (NASH), are associated with insulin resistance and the metabolic syndrome, and approximately 70% of patients with diabetes mellitus type 2 (T2DM) commonly develop NAFLD [50,103]. Moreover, NAFLD patients manifest a high prevalence of insulin resistance [104], and approximately 80% of NAFLD patients are obese, although NAFLD may also occur in lean patients. 

Hepatic steatosis in NAFLD is associated with elevated de novo lipogenesis with reduced VLDL-triglyceride output, and a surplus of free fatty acids (FFAs) in association with peripheral insulin resistance and the release of inflammatory chemokines, eicosanoids, and mitochondrial dysfunction, and is, therefore, considered the hepatic manifestation of metabolic syndrome [103,105]. However, owing to a paucity of animal models mimicking human NAFLD, the relationship between insulin resistance and NAFLD remains a challenging hen-and-egg question. This is rooted in the observation that NAFLD is emerging from multiple parallel hits and involves inflammation, lipotoxicity, insulin resistance, and gut leakiness and dysbiosis [106,107,108,109,110]. Inflammatory processes that initiate NAFLD-associated inflammation and progression to NASH can reside in the liver as well as in extrahepatic tissues, such as white adipose tissue (WAT) and the gut (see previous section). NAFLD may progress to NASH, a chronic and irreversible inflammatory complication of NAFLD. Hence, it is important to identify inflammatory triggers that mediate inflammation in NAFLD and pave the way to NASH progression. Subsequently, chronic hepatitis in NASH can induce progression into liver fibrosis and cirrhosis and predisposes to hepatocellular carcinoma. NASH is characterized by macrovesicular steatosis, inflammatory infiltrates, hepatocyte ballooning, and variable degrees of characteristic chicken-wire fibrosis. NASH has become the overall second leading cause for liver transplantation in the USA, and in white females, NASH has lately outnumbered alcoholic liver disease and chronic hepatitis C infection as a leading cause for transplant [111]. Currently, 25% of the world population are estimated to exhibit NAFLD [107]. However, the incidence of NASH varies among different ethnicities, and is also modulated by genetic variations, lifestyle, diet and the composition of the gut microbiome [105,111], among other environmental factors. 

Physiologically, portal insulin level exceeds that in the peripheral circulation by about threefold [112]. This is consistent with the higher transcriptional level of lipogenic genes (such as fatty acid synthase-FASN) in hepatocytes due to the activation of sterol responsive element binding protein 1c (SREBP1c) transcription factor [113]. Despite the higher level of lipogenic genes in the liver, the activity of FASN in this tissue is almost undetectable under normal insulinemic conditions to minimize de novo lipogenesis. This “protective” mechanism is mediated by the pulsatility of insulin release from pancreatic β cells [114] that acutely activates the insulin signaling pathways and phosphorylation of CEACAM1 in hepatocytes, which in turn facilitates endocytosis of insulin and its targeting to the degradative pathways. Concurrently, this leads to binding of CEACAM1 to FASN and suppression of its activity [115]. In this manner, by promoting appropriate insulin clearance [114], CEACAM1 mediates insulin sensitivity and restricts de novo lipogenesis in liver under normal insulinemic conditions. Under chronic hyperinsulinemia, such as when insulin clearance is impaired, the pulsatility of insulin secretion is diminished [112], resulting in blunted acute insulin signaling, hepatic insulin resistance and limited counter-regulation of lipogenesis. Together, this enhances hepatic lipid production and favors re-esterification over fatty acids β-oxidation causing hepatic steatosis in addition to lipid redistribution to visceral adipose depots to be stored [105,116,117]. Eventually, lipolysis occurs and drives the flow of toxic FFAs from visceral fat into the liver (and other tissues) where they substantiate ectopic fat accumulation and cause systemic insulin.

In addition to FFAs release, adipose-tissue-infiltrating bone-marrow-derived cells differentiate into proinflammatory M1-type macrophages that release adipokines and chemokines to contribute to systemic insulin resistance [104,107], as exemplified by the suppressive effect of TNFα on insulin signaling [103,118]. In addition to adipokines, disturbance of the gut permeability barrier enhances the penetration of pathogen-associated molecular patterns (PAMPs) and microbe-associated molecular patterns (MAMPs) into the circulation that pre-program liver-resident immune cells, such as Kupffer cells and T cells, to undergo inflammation and facilitate loss of tolerance [105,119,120]. Steatohepatitis in NASH involves a Th1 cytokine response that drives liver injury and elevated levels of proinflammatory cytokines, like TNFα, IL-1β, IL-6, interferon γ (IFNγ) and first apoptosis signal (Fas)-ligand are detected in serum, liver and adipose tissue of humans and mice with NASH phenotype [121]. Their relative levels correlate with disease severity, and especially elevation of TNFα, as bolstered by reduced steatohepatitis, apopotosis and fibrosis by the anti-TNFα antibody Infliximab^TM^ [104,122]. 

**Figure 4:**
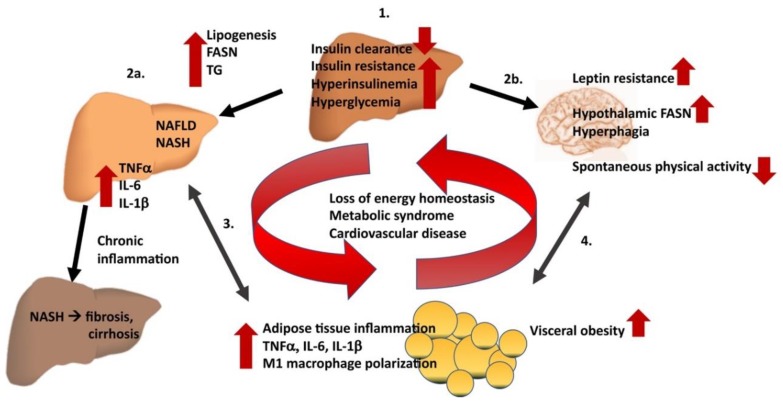
Pathophysiological consequences of loss of CEACAM1 expression and function. Hepatic inactivation or global loss of functional CEACAM1 expression impairs insulin clearance to cause insulin resistance and fatty liver disease in addition to disturbed energy homeostasis. Initially, these *Ceacam1* mutations impair insulin clearance to promote hyperinsulinemia and insulin resistance (1). Hyperinsulinemia, in turn, promotes lipogenesis in the liver, accompanied by elevated fatty acid synthase (FASN) expression and activity, followed by hepatic fat accumulation (2a). Subsequently, NAFLD and NASH emerge, accompanied by elevation of inflammatory cytokine production (TNFα, IL-6, IL1β). The emergence of macrosteatosis as well as fibrosis can be accelerated by high-fat-diet feeding of *Ceacam1* mutant mice. Chronic liver inflammation can ultimately lead to more progressive liver fibrosis and cirrhosis. Paralleling insulin resistance, hyperinsulinemia, fatty liver disease and disturbed energy homeostasis, visceral adiposity and systemic inflammation emerge (3). Additionally, leptin resistance, elevation of hypothalamic FASN expression, hyperphagia and reduction of energy expenditure (2b/4) fuel the vicious cycle that ultimately contributes to the cardio-metabolic abnormalities in *Ceacam1*-deficient mice. Upward or downward red arrows indicate increase or decrease, respectively, in cardio-metabolic or inflammatory events/parameters. Single-headed black arrows indicate hierarchical chains of events in the emergence of metabolic dysfunction. Double-headed black arrows depict feedback loops pointing towards the connection between visceral obesity and adipose tissue inflammation on the one hand and hepatosteatitis (3.) and central control circuits in obesity (4.) on the other.

Obese insulin-resistant humans with NAFLD exhibit reduced levels of hepatic CEACAM1 in a linear relationship with the severity of the disease [123]. Similarly, primary hepatocytes from obese humans and rat models of obesity and hepatic steatosis exhibit a decline in CEACAM1 levels [117]. Experiments in mouse models targeting CEACAM1 expression globally or in the liver shed more light on how hepatic CEACAM1 exhibits unique regulatory properties connecting insulin clearance and lipid metabolism to the pathogenesis of NAFLD/NASH. Global null deletion of the *Ceacam1* gene and liver-specific inactivation of CEACAM1 impairs insulin extraction to cause hyperinsulinemia, followed by insulin resistance and secondary increase in hepatic lipid production and redistribution to the white adipose tissue to yield visceral adiposity and lipolysis [31,101,117,124,125,126,127,128,129] (Figure 4). 

Hyperinsulinemia and corruption of CEACAM1-mediated repression on hepatic fatty acid synthase (FASN) activity in hyperinsulinemia further support hepatic steatosis and SREBP1c-mediated lipogenesis and are accompanied by a reduction in hepatic β-oxidation [115,127,130]. Furthermore, global *Ceacam1* null mice develop leptin resistance, which contributes to hyperphagia, fat accumulation, and reduction of physical inactivity; all leading to obesity. Moreover, hyperinsulinemia in *Ceacam1^−/−^* mice induces hypothalamic FASN level and activity, which contributes to hyperphagia and physical inactivity [131,132,133]. Furthermore, fatty acid β-oxidation is inhibited in muscle of *Ceacam1^−/−^* mice, which enhances triglyceride accumulation [131]. Conversely, exclusive transgenic hepatic restoration of functional CEACAM1-L reversed all of the metabolic abnormalities caused by global *Ceacam1* deletion in *Ceacam1^−/−/liver+^* mice with exclusive transgenic liver-specific CEACAM1 restoration [129]. This includes hyperinsulinemia, insulin resistance, and steatohepatitis. Moreover, FASN activity in the hypothalamus was repressed, contributing significantly to the normalization of hyperphagia [129].

That obesity and hepatic steatohepatitis are associated with impaired insulin clearance is demonstrated by the repression of hepatic CEACAM1 expression by high-fat (HF) intake [134] via a peroxisome-proliferator-activated receptor α (PPARα)-dependent mechanism [135]. Forced liver-specific transgenic overexpression of CEACAM1 protected the liver [134] and white adipose tissue [136] against diet-induced metabolic, inflammatory and fibrogenic response [134]. Adenoviral-mediated reintroduction of intact wild-type CEACAM1 in hepatocytes protects against the adverse negative metabolic effects of a high-fat (HF) diet: These include hyperinsulinemia, hepatic lipid production and steatohepatitis [134,136]. Similarly, treatment of HF-fed wild-type mice with exenatide, a long-acting glucagon-like peptide-1 receptor agonist that stimulates insulin secretion, prevented diet-induced insulin resistance and altered metabolism by restoring hepatic CEACAM1 expression via a PPARγ-dependent pathway [137]. Exenatide treatment also protected against diet-induced steatohepatitis [137,138] and activation of the pro-fibrogenic TGFβ-Smad2/3 signaling pathway [138]. Collectively, these studies emphasize the importance of the regulation of insulin sensitivity by homeostatic insulin levels, which are in turn, regulated by insulin secretion from pancreatic β cells as well as by its hepatic extraction.

### 2.5. CEACAM1: Regulating a Cross-Talk Between the Liver and the Cardiovascular System

In addition to insulin resistance and hepatosteatosis, *Ceacam1*^−/−^ mice develop endothelial dysfunction, as well as cardiac hypertrophy with septal wall thickening, reduction in cardiac performance and disturbance of vasomotor activity, elevation of cardiac and aortic lipid content, and increased oxidative stress and apoptosis [92,139]. Endothelial dysfunction that is caused by insulin resistance, dyslipidemia, and hyperglycemia in *Ceacam1^−/−^* mice causes a reduction in endothelial nitric oxide (NO) levels due to impaired endothelial activation of IRS1/Akt/eNOS (endothelial nitric oxide synthase pathway), in addition to an imbalanced endothelin-1 (ET-1) receptor A/B expression [139]. Furthermore, insulin signaling in heart tissue is compromised owing to a hyperinsulinemia-driven reduced insulin receptor number [139]. Liver-specific rescuing, however, reversed these abnormalities in *Ceacam1^−/−−/liver+^* mice, normalizing plasma NO and ET-1 levels and preventing leukocyte adhesion to the aortic walls [139]. These data indicate that hepatic CEACAM1 expression is necessary and sufficient to maintain insulin and metabolic homeostasis, and to protect against insulin resistance and its consequences, as well as cardiovascular disease elicited by metabolic inflammation. 

### 2.6. CEACAM1: Implication in Mucosal and Hepatic Immune Regulation

CEACAM1 is expressed in T, B, NK, and dentritic cells, and in granulocytes, monocytes and macrophages [26,31,140,141,142,143,144,145,146,147]. In naïve lymphocytes, CEACAM1 is expressed at low levels, but undergoes a rapid upregulation upon cellular activation. In contrast, in granulocytes, CEACAM1 is a differentiation antigen that controls granulopoiesis and delays neutrophil apoptosis [148,149]. CEACAM1 is generally associated with protection against hyperinflammation that results from either inappropriate expansion of cellular precursors such as in dysregulated granulopoeiesis and neutrophilia in *Ceacam1^−/−^* mice, associated with enhanced IL-1β production in response to stimulation of toll-like receptor 4 (TLR4) or exaggerated effector T cell responses or dysfunctional B cell activation and survival [91,148,150,151]. 

In the regulation of immune cells by CEACAM1, its ligand binding properties, either as a monomer or a homo- or heterodimer, play a pivotal role. In addition to its homophilic interaction, CEACAM1 can bind to other members of the CEA family (such as CEACAM1-5,6, or 8 in humans) in a heterophilic fashion. CEACAM1 also serves as a receptor for a vast variety of pathogens in both humans and mice (in humans: *N. gonorrhoe* and *N. meningitidis*, *H. influenzae*, *M. catharalis*, *H. pylori*, *E.coli*, *Salmonellae spp.*, *Candida albicans* and the hepatitis virus A59 (MHV) in mice [21,152,153,154,155,156,157,158,159,160,161,162,163,164,165,166], and Horst, A.K. and Wagener, C.; unpublished observations). Pathogen binding or homomeric or heteromeric interaction between CEACAMs occur predominantly via their N-terminal domain [167,168,169,170,171]. In mice, CEACAM3,5,6 and 8 are not expressed [29]. The relative expression density of *Cis* homo- and heterodimers and isomers with either a long or short cytoplasmic domain determines the signaling outcome by CEACAM1 and provides the molecular basis for complex signal integration. CEACAM1-mediated effects on cellular activation, proliferation and cytokine production are controlled by several factors. These include: (i) Its overall expression levels and subcellular localization; (ii) the spatiotemporal upregulation and expression of CEACAM1 isoforms that are expressed on a cognate immune cell or tissue; (iii) the relative ratio between the major isoforms (L vs. S.), and (iv) by self-ligation or heteroligation in *cis* or *trans*, either with CEACAM1 molecules or other co-receptors on the same or a neighboring cell [5,172]. Furthermore, binding of pathogen-derived heteroligands can disrupt homodimeric CEACAM1 interactions and thereby modulate downstream signaling [157]. 

### 2.7. CEACAM1-Dependent Regulation of T Lymphocyte Activation

Since homeostasis in the T cell compartment plays a pivotal role in the etiology of autoimmune liver disease and immune-mediated liver injury, the focus in this section will be set on CEACAM1-mediated control in T cell activation and the induction of regulatory T cells and its implications in hepatic and mucosal immunity of the gastrointestinal tract.

CEACAM1 is the only member of the CEA family that is expressed on activated CD4^+^ and CD8^+^ T cells, where it is rapidly upregulated upon TCR stimulation [140,142,173]. In naive CD4^+^ T cells, CEACAM1 is expressed at very low levels, and is stored intracellularly in endosomal compartments [173]. In activated T cells, CEACAM1 shows overlapping expression kinetics with the activation marker CD69 and precedes the expression of cytotoxic T-lymphocyte-associated protein 4 (CTLA-4) [173,174]. This indicates that the inhibitory form, CEACAM1-L, can interfere with T cell activation prior to CTLA-4, and is therefore, an independent immune checkpoint regulator. CEACAM1-S and CEACAM1-L exert opposite roles in the control of T cell activation with CEACAM1-S acting as an independent T cell activator from T cell receptor (TCR) stimulation that triggers cytokine production (IL-2, IFNγ) in response to IL-2, IL-7 and IL-15, as well as up-regulation of the Ca2^+^-dependent nuclear factor activator of transcription (NFAT)-mediated signaling. NFAT signaling propagates expression of IL-2, IL-4, IFNγ, and CD40L [140,142,152,175,176]. In contrast, CEACAM1-L controls T cell activation by inhibiting downstream signaling pathways [5,66,177]. As mentioned above, it contains ITIM/ITSM motifs that are phosphorylated by Src-like kinases like p56^Lck^ in T cells to mediate binding to SHP-1, SHP-2 and blunting of downstream kinase-dependent pathways. These include activation of the CD3ζ chain, the TCR-associated protein kinases ZAP-70, mitogenic and proinflammatory kinases, extracellular signal-regulated kinases (ERK), and Jun-activated kinases (JNK) [177,178,179]. A recent report demonstrated that recruiting of p56^Lck^ to the TCR-signaling complex in CD8^+^ T cells depends on CEACAM1-L expression and its binding to filamin A, which stabilizes the immunological synapse and supports CD8^+^ T cell proliferation to prevent their exhaustion in viral infection [180,181]. In addition, CEACAM1-L can interfere with viral replication in human cytomegaly virus infection via SHP-2-mediated downregulation of molecular target of rapamycin (mTOR) activation [182]. Furthermore, CEACAM1-L can associate with the β chain of the IL-2 receptor (IL-2Rβ) and modulates ZAP-70-signaling and SHP-1 binding that are normally required for T cell-mediated cytolytic function that is elicited after TCR activation. Blockage of CEACAM1 homophilic binding suppresses cytolytic function elicited by TCR-signaling [179]. Additionally, CEACAM1-L interacts with β-catenin that regulates Fas-induced apoptosis in Jurkat cells [183]. 

T cell activation also largely depends on the kinetics of CEACAM1 isoform expression on T cells, and substantial evidence exists to support the notion that CEACAM1-L acts as an inhibitory receptor on T cell activation, whereas the CEACAM1-S isoform is an independent T cell activator [176]. During mucosal inflammation, such as in models of inflammatory bowel disease provoked by dextran sodium sulfate (DSS), trinitrobenzine sulfonic acid and oxazolone painting, both heterophilic ligation of CEACAM1 with antibodies or homophilic ligation by soluble recombinant CEACAM1-Fc fusion proteins inhibits mixed lymphocyte reactions in vitro, and hapten-mediated colitis in vivo, and is associated with inhibition of the production of proinflammatory Th1 cytokines via T-bet signaling, but not STAT4-promoted Th2 T cell differentiation and signaling [172,173,184,185,186]. Hence, *Ceacam1^−/−^* mice exhibit a higher production of pro-inflammatory Th1 cytokines due to a lack of CEACAM1-L expression. Interestingly, ligation of CEACAM1 with MHV spike proteins blocks differentiation towards the Th1 type [184]. In addition, CEACAM1 can also convey tolerance by interaction with the immune checkpoint regulator T cell immunoglobulin domain and mucin domain-3 (TIM-3) protein, which is an activation-induced inhibitory co-receptor. TIM-3 is co-expressed with CEACAM1 during tolerance induction during chronic viral infection in inflammatory bowel disease (IBD) and in colon tumors. It associates with CEACAM1 via its extracellular domains as a heterodimer in both *Cis* and *Trans*, and this interaction is required to enhance TIM-3 cell surface expression and maturation, as well as promoting its inhibitory functions [187,188]. In this manner, CEACAM1 expression on T cells can convey tolerance by interaction with another immune co-receptor. However, as described below, CEACAM1 can also adopt tolerogenic properties by propagating regulatory T cell induction and stability, depending on the contextual expression of the CEACAM1 variant with the short cytoplasmic isoform. Indeed, besides the generally accepted inhibitory function of the CEACAM1-L isoform, the expression of CEACAM1-S can control T cell-borne inflammation via eliciting regulatory T cells.

### 2.8. Gastrointestinal and Hepatic T Cell Activation and Regulatory T Cell (Treg) Induction Depend on Spatiotemporal Expression of Both Ceacam1 Isoforms

In addition to the extracellular interaction between TIM-3 and CEACAM1 in the induction of tolerance via mediating immune-inhibitory signals, mucosal immunity is affected by specific CEACAM1 isoform expression in the gut. As described above, another dimension to the complex regulatory network in fine-tuning immune regulation via CEACAM1 is added by the observation that there is a tissue-specific predominance of CEACAM1-L or CEACAM1-S isoform expression [176]. In the intestines and gut-associated lymphoid tissues, predominance of the T cell-associated CEACAM1-S isoform is found in humans and mice, compared to CEACAM1-L. In contrast, CEACAM1-L is the major CEACAM1 isoform expressed in T cells in extra-intestinal tissues [5,176]. This is important to note since CEACAM1-S-dependent T cell activation is important to elicit T cell subset education into TGFβ-expressing CD4^+^latency peptide^+^ (LAP) T cells, which can induce mucosal immunity-enhancing follicular T helper (Tfh) cells that trigger the production of secreted IgA by follicular B cells in mice. This secretory mucosal immunity is elicited upon gastrointestinal bacterial antigen exposure, as demonstrated after challenging the wild-type, *Ceacam1^−/−^*, and *Ceacam1-S* transgenic mice with overexpression of CEACAM1-S in their CD4^+^ T cells with *L. monocytogenes* [176]. The LAP^+^-expressing CD4^+^CD25^−^ and CD4^+^CD25^+^ Treg populations from mesenteric lymph nodes are especially interesting, since they appear to exhibit unique immune suppressive capacities that are mediated by TGFβ [189]. Of note, these cells can be induced by oral feeding of CD3 antibodies and can suppress experimental autoimmune encephalitis (EAE) [189]. Intravenous injection of anti-CD3 antibodies did not produce this effect and induced apoptosis after T cell activation, thus leading to T cell deletion [189]. In the model of *L. monocytogenes* infection, *Ceacam1^−/−^* mice failed to substantially expand the CD4^+^CD25^−^LAP^+^ population upon oral feeding of anti-CD3 antibodies but did not exhibit alterations in their CD4^+^Foxp3^+^ Treg pool in the gastrointestinal-associated lymphatic tissues [176].

In addition, mice expressing or over-expressing CEACAM1-S in their CD4^+^ T cell compartment exhibited increased diversity of their gut microbiota compared to controls. The predominance of the CEACAM1-S isoform requires persistent exposure to mucosal antigens, which is required to confer robustness towards pathogenic enteropathogens, such as *L. monocytogenes* [176]. Hypersensitivity towards *L. monocytogenes* has also been reported in *Ceacam1^−/−^* mice as a result of dysregulation in granulopoiesis in the absence of CEACAM1-L in the myeloid compartment, in addition to neutrophilia resulting from impaired inhibition of Stat3 activation downstream of the G-CSF receptor that controls granulopoiesis. This, in turn, leads to septicemia after *L. monocytogenes* infection with dramatic elevation of IL-1β, TNFα, and IL-6 [148]. In addition, CEACAM1 acts as a prominent receptor for commensal and pathogenic gastrointestinal microbes (see above) that can modulate gut-associated immunity and systemic tolerance by release of bacterial metabolites. These are instrumental in mediating liver tolerance via induction of T cell populations that migrate from the intestines and suppress inflammation in the hepatic immunological environment [190,191,192,193,194]. The gut-liver axis and aberrant gut permeability are important determinants in the emergence of metabolic dysfunction that cause or accompany liver inflammation in the metabolic syndrome, obesity, and NAFLD/NASH (see below). 

### 2.9. CEACAM1 Is Required for Treg Induction in the Liver and Protects against Immune-Mediated Hepatitis in Mice

In addition to mucosal immunity, we have recently demonstrated that Treg induction and stability in the liver is also controlled by CEACAM1 expression in murine T cells [195]. The connection between the gut and the liver can contribute significantly to extraintestinal manifestations of IBD, and vice versa, either by atypical immune cell homing of mucosal T cells, or by persistence and relocation of self-reactive T cells to the liver [196]. However, immune-mediated liver inflammation and IBD can occur independently of each other and often appear disconnected in their relative time courses. Circulation of mucosal T cells via the thoracic duct enables immunological cross-talk between the gut and the liver, and oral tolerance is compromised by the introduction of a portacaval shunt [190,197]. Yet, it is unknown whether mucosal T cells could travel to the liver to import tolerogenic or inflammatory signals. 

Primary biliary cirrhosis (PBC), primary sclerosing cholangitis (PSC) and autoimmune hepatitis (AIH) are autoimmune diseases that share the common hallmark of compromised Treg homeostasis and often hyperexpansion with enhanced pro-inflammatory activity of effector T cells, which is also observed in type 1 diabetes, rheumatoid arthritis and graft-versus host-disease. Treg are instrumental in the upkeep of peripheral tolerance by mediating immunosuppression exerted by TGFβ- or IL-10-dependent immune suppression, granzyme A- or B-dependent killing of target T effector cells, competition for microenvironmental IL-2 pools, or inhibition of dendritic maturation and function [198,199]. Cell-to-cell contact is required for Tregs to exert their suppressive function in vitro [200]. IL-2 controls the induction expansion of regulatory T cells and effector T cells via binding to high or low affinity IL-2 receptors. On Tregs, the high affinity receptor CD25 (the IL-2Rα chain) in the trimeric high affinity IL-2 receptor, is expressed in high levels and ensures efficient binding of IL-2 even if this cytokine is present in the microenvironment in very low amounts [201]. IL-2 is largely produced by effector T cells, and preferred IL-2 consumption by Treg ensures their expansion. Treg function and survival depends on IL-2, and IL-2R-mediated phosphorylation of signal transducer and activator of transcription 5 (STAT5), its nuclear translocation and subsequent Foxp3 and CTLA-4 expression [202,203,204,205,206]. To date, it has been shown that therapy with low doses of IL-2 is effective against autoimmune diseases, such as lupus erythematodes, type 1 diabetes, rheumatoid arthritis, and graft-versus host disease [207,208,209,210,211,212] partially by inducing endogenous expansion of Tregs in human patients [204,213,214]. The importance of this approach is emphasized by the observation that polymorphisms of the human IL-2RA result in reduction of CD4^+^Foxp3^+^ Tregs and their suppressive function in patients with primary sclerosing cholangitis [215]. A novel antibody has been developed that allows selective targeting and expansion of Tregs by preferentially inducing STAT5 phosphorylation [216]. 

**Figure 5:**
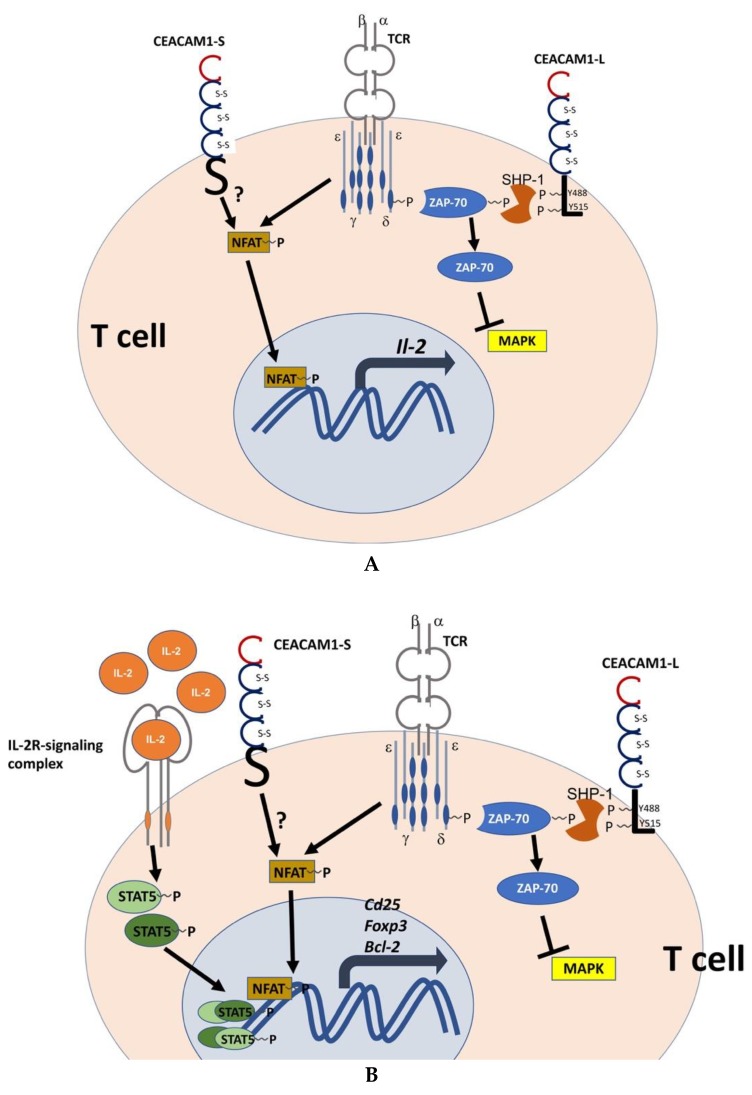
Specific functions of CEACAM1-S and CEACAM1-L isoforms in the regulation of T cell activation. (**A**) Different features of the CEACAM1-isoforms, CEACAM1-S and CEACAM1-L, in T cell signaling: CEACAM1-S and the TCR complex trigger T cell activation, and NFAT-mediated signaling [176,231]. Subsequently, NFAT nuclear translocation and transcription of *Il-2* are initiated; CEACAM1-S can act as an independent signaling unit in T cells. CEACAM1-L with two ITIM motifs is phosphorylated by Src family kinases, and subsequently binds SHP-1 that dephosphorylates ZAP-70 and consequently blocks TCR-mediated activation of MAPK signaling in T cells [5,177,178]. The molecular adaptors that relay CEACAM1-S signaling via NFAT1 have not been defined yet. The initiation of CEACAM1-S-mediated IL-2 production is a key event in Treg induction and stability (**B**). Upon CEACAM1-S and TCR-induced IL-2 production, signaling of the IL-2R complex induces STAT5 phosphorylation; phosphorylated STAT5 translocates into the nucleus and acts as a transcription factor in a heterodimeric or heterotetrameric complex. pSTAT5 controls the transcription of key functional Treg genes, such as *CD25*, *Foxp3*, and *Bcl-2*, indicated by a curved black arrow ([204], and references therein)*.* Single-headed black arrows indicate a chain of downstream phosphorylation and dephosphorylation events: Phosphorylation is elicited by the IL-2R, as well as as TCR in conjuction with CEACAM1-S and CEACAM1-L; in turn, SHP-2 recruitment by phosphylated CEACAM1-L localizes ZAP-70 phosphatase to TCR, which consequently blocks MAPK activation (black T bar). The question mark downstream of CEACAM1-S indicates that direct activation and phosphorylation of NFAT by CEACAM1-S via potential physical interactions remains to be determined. Reprinted with adaptations from [195] with permission from Wiley and Sons.

As discussed above, CEACAM1-L regulates TCR- and IL-2R-mediated signaling. However, evidence that the short CEACAM1 isoform can similarly respond to IL-2R signaling with specific impact on T cell polarization has not been described. In our mouse model of immune-mediated hepatitis that reflects several aspects of T cell activation including cytokine response observed in autoimmune hepatitis [Concanavalin A (ConA) hepatitis, [217,218,219]], we have demonstrated that induction of intrahepatic regulatory T cells depends on IL-2 production by CEACAM1^+^CD4^+^ T cells, as IL-2 production was significantly reduced in CEACAM1^−^CD4^+^ T cells [195]. ConA binding to the liver sinusoidal endothelium [217,220] activates liver resident immune cells and triggers a cascade of inflammation that is initiated by the production of IFNγ and TNFα by effector T cells and monocytes/macrophages, respectively [217,218,221]. Subsequently, hepatocytes undergo TRAIL-dependent cell death [222,223] thereby further enhancing hepatitis by the release of damage-associated molecular patterns (DAMPs) and other alarmins [224] which exacerbate liver injury and inflammation by the activation of innate lymphoid and myeloid cells [218,225,226]. *Ceacam1^−/−^* mice show a more severe and extended course of ConA hepatitis, with significantly elevated liver enzymes and hepatic necroinflammation. Furthermore, analysis of their intrahepatic T cell populations showed a reduction in intrahepatic CD4^+^Foxp3^+^ Tregs, concurrent with hyperexpansion of CD4^+^ Th1 cells [195]. This phenotype was also transmittable by the transfer of CEACAM1^−^CD4^+^ T cells into an immune-compromised host strain. In extended analyses addressing CEACAM1 isoform expression in CD4^+^ T cells after ConA challenge, we found upregulation of CEACAM1-L in CD4^+^CD25^−^ cells, which is crucial to dampen T cell-borne hyperinflammation. In contrast, in CD4^+^CD25^+^ T cells, we observed upregulation of CEACAM1-S. Hence, initial IL-2 production by CEACAM1^+^CD4^+^CD25^+^ effector T cells is essential to induce appropriate CD4^+^CD25^+^Foxp3^+^Treg cells in the liver, since CEACAM1-S expression enhances signaling via the IL-2 receptor and induces phosphorylation of STAT5, the Treg master regulator transcription factor [195]. Activation of STAT5 is required for its nuclear translocation, where it binds to the promoters of the IL-2 receptor (CD25), Foxp3, and the Treg survival factor Bcl-2 and, therefore, fosters induction and stability of Tregs [206]. In CEACAM1^−^CD4^+^ T cells, STAT5 phosphorylation is impaired [195]. In addition, CEACAM1-L interacts with the IL-2Rβ and the common cytokine receptor γ chain, but not with the high-affinity IL-2Rα chain (CD25). To date, however, it is unknown whether CEACAM1-S can associate with CD25 and stabilize its expression on the T cell surface or modulate the affinity of IL-2 to its receptor by sterical interaction with CD25. Additionally, CEACAM1-expressing Tregs could compete for local pools of IL-2, and subsequently induce apoptosis in neighboring T effector cells by IL-2 deprivation [227,228]. In this respect, it is important to note that T cells (Jurkat cells) undergo First apoptosis signal (Fas)-dependent apoptosis after activation [183]. The induction of apoptosis depends on the interaction between CEACAM1-L long cytoplasmic domain and β-catenin and can be ablated by mutagenesis of critical residues within Armadillo repeats [183,229]. Hence, activation-induced apoptosis may be impaired in CEACAM1^−^CD4^+^ Th1 cells. Another negative regulator feedback loop that is missing in CEACAM1-deficient cells comprises the induction of CEACAM1 expression by IFNγ [230]. Taken together, these data described the dual role of CEACAM1 and its two major isoforms in T cell activation and polarization. Although the net outcome of CEACAM1-dependent T cell regulation is to dampen inflammation, the initial inflammatory cascade is triggered by TCR-dependent upregulation of the stimulatory isoform CEACAM1-S in activated CD4^+^CD25^+^ cells, which is required to induce TCR-dependent nuclear factor of activated T cells (NFAT) activation and IL-2 production and subsequent STAT5-dependent signaling pathways to confer Foxp3^+^ expression and Treg induction [195]. On the other hand, hyperactivation of Th1 effector cells is prevented by upregulation of CEACAM1-L in the CD4^+^CD25^−^ population, but not in the CD4^+^CD25^+^Treg population. These studies demonstrated that CEACAM1 isoform expression controls T cell homeostasis and the maintenance of tolerance by spatiotemporal regulation of CEACAM1 isoform expression in the liver [195], as depicted in (Figure 5A,B). However, whether CEACAM1-S could assist in conformational stabilization of IL-2 binding to the IL-2 receptor, or whether CEACAM1-ligation by soluble CEACAMs or antibodies could potentially support Treg expansion and enhance their suppressive function in autoimmune liver diseases, remains to be demonstrated. 

## 3. Conclusions

CEACAM1 can adopt dual roles in tissue differentiation, cellular proliferation, motility, and signaling that are fine-tuned by a cell- and tissue-specific relative distribution of its CEACAM1-L and CEACAM1-S isoforms. Hence, CEACAM1 can both support or inhibit tissue differentiation, tumorigenesis and metastasis. Moreover, self-ligation in *Cis* and *Trans* as well as heterophilic binding to other CEACAMs, microbial adhesins or growth factor receptor tyrosine kinases and immune checkpoint regulators control CEACAM1-mediated effects on cellular signaling. CEACAM1 expression is generally up-regulated in inflammation, and it can exert inhibitory and activating signals by temporal control of its long and short isoform expression. To date, very few disease-associated polymorphisms or mutations of CEACAMs have been described in humans, indicating that this family of adhesion molecules fulfills non-redundant functions in immunity, cellular differentiation, and metabolic regulation [232,233]. However, dysregulation of CEACAM expression is commonly observed in a variety of tumors. By selective isoform expression in a tissue-specific manner, CEACAM1-S can contribute to the generation of regulatory T cells and organ protection in the gastrointestinal tract and liver which are organs with a central role in the upkeep of systemic tolerance. For instance, CEACAM1-S expression in CD4^+^ T cells is essential to elicit protection from mucosal infection and immune-mediated liver injury via Treg induction, whereas CEACAM1-L expression in T effector cells and neutrophils dampens inflammation. In addition, CEACAM1 controls insulin clearance to protect against insulin resistance, obesity, WAT-associated inflammation, hepatosteatosis (NAFLD), NASH, fibrosis and cardiovascular disease, as bolstered by observations in mice with global deletion of CEACAM1 expression. It is remarkable that rescuing hepatic expression in a *Ceacam1*-deficient background suffices to restore metabolic homeostasis even under high-fat diet intake. The ubiquitous expression of CEACAM1 in humans, however, poses a substantial challenge for the development of CEACAM1-directed therapeutics in order to prevent adverse side-effects, which will require in-depth mechanistical future studies.

## Abbreviations

AIH	Autoimmune hepatitis
ALP	Alkaline phosphatase
AP1, AP2	Adapter protein 1, 2
BGP-I	Biliary glycoprotein-I
C-CAM	Calcium-dependent cell-cell adhesion molecule
CD	Cluster of differentiation
CEACAM1	Carcinoembryonic antigen-related cell adhesion molecule 1 protein in humans and rodents
*Ceacam1*	Gene encoding CEACAM1 protein in mice
*CEACAM1*	Gene encoding CEACAM1 protein in humans
DSS	Dextran sodium sulfate
EAE	Experimental autoimmune encephalitis
eNOS	Endothelial nitric oxide synthase
ERK	Extracellular signal-regulated kinases
ET-1	Endothelin-1
Fas	First apoptosis signal
FASN	Fatty acid synthase
FFAs	Free fatty acids
Foxo1	Forkhead box protein O1
GGT	γ Glutaryl transferase
Grb2	Growth factor receptor-bound protein 2
HCC	Hepatocellular carcinoma
HF	High-fat diet
HNF	Hepatocyte nuclear factor
IBD	Inflammatory bowel disease
IFNγ	Interferon γ
IGF-1	Insulin-like growth factor 1
IGFR	Insulin-like growth factor receptor
IR	Insulin receptor
IRS-1	Insulin receptor substrate-1
ITIM	Immunoreceptor tyrosine receptor-based inhibition motif
ITSM	Two immunoreceptor tyrosine-based switch motifs
JNK	Jun-activated kinases
L-SACC1	Mice with transgenic liver-specific overexpression of the phosphorylation-deficient dominant-negative Ser503Ala mutant under the control of apolipoprotein A1 promoter
LAP	Latency peptide
LD	Linear dichroism
LIRKO	Mice with conditional null mutation of the IR in the liver
MAMPs	Microbe-associated molecular patterns
MHVR	Murine hepatitis virus receptor
NAFLD	Non-alcoholic fatty liver disease
NASH	Non-alcoholic steatohepatitis
NFAT	Nuclear factor of activated T cells
PAMPs	Pathogen-associated molecular patterns
PBC	Primary biliary cirrhosis
PDIP38	Polymerase delta interacting protein p38
PPARα	Peroxisome-proliferator-activated receptor α
PSC	Primary sclerosing cholangitis
β2SP	Smad (small mothers against decapentaplegic) adaptor protein
Shc	SH2-containing adapter protein
SHP	SH2-containing tyrosine phosphatase
Smad	Small mothers against decapentaplegic
SREBP1c	Sterol responsive element binding protein 1c
STAT	Signal transducer and activator of transcription
TGFβ	Transforming growth factor β
TIM-3	T cell immunoglobulin domain and mucin domain-3
TLR4	Toll-like receptor 4
Treg	Regulatory T cell
WAT	White adipose tissue
